# Systemic interrogation of immune-oncology-related proteins in patients with locally advanced prostate cancer undergoing androgen deprivation and intensity-modulated radiotherapy

**DOI:** 10.1007/s00345-024-04787-8

**Published:** 2024-02-22

**Authors:** Ingrid Jenny Guldvik, Håkon Ramberg, Gitte Kristensen, Andreas Røder, Ian G. Mills, Wolfgang Lilleby, Kristin Austlid Taskén

**Affiliations:** 1 Department of Tumor Biology, Institute for Cancer Research, Oslo University Hospital, Oslo, Norway; 2 Institute of Clinical Medicine, University of Oslo, Oslo, Norway; 3 Department of Urology, Center for Cancer and Organ Diseases, Copenhagen Prostate Cancer Center, Copenhagen University Hospital - Rigshospitalet, Copenhagen, Denmark; 4 Department of Clinical Medicine, University of Copenhagen, Copenhagen, Denmark; 5 Cancer Research UK, Li Ka Shing Centre, Cambridge Research Institute, Cambridge, UK; 6 Nuffield Department of Surgical Sciences, John Radcliffe Hospital, University of Oxford, Oxford, UK; 7 Patrick G. Johnston Centre for Cancer Research, Queen’s University of Belfast, Belfast, UK; 8 Department of Oncology, Oslo University Hospital, Oslo, Norway

**Keywords:** Biomarkers, Hormone treatment, Noninvasive, Leucine-rich alpha-2-glycoprotein 1, LRG1, Prostate cancer, Intensity-modulated radiotherapy, Treatment resistance

## Abstract

**Purpose:**

The primary objective was to establish whether blood-based leucine-rich alpha-2-glycoprotein (LRG1) can predict outcomes in patients with locally advanced prostate cancer undergoing androgen-deprivation therapy (ADT) and radiotherapy (RT) and to determine how it may relate to 92 immune-oncology (I-O)-related proteins in this setting.

**Methods:**

Baseline blood level of LRG1 from patients treated with ADT and RT enrolled in the CuPCa (*n* = 128) and IMRT (*n* = 81) studies was measured using ELISA. A longitudinal cohort with matched blood samples from start of ADT, start of RT, and end of RT protocol from 47 patients from the IMRT cohort was used to establish levels of I-O proteins by high-multiplexing Proximal Extension Assay by Olink Proteomics.

Statistical analyses using Kaplan–Meier, Cox regression, and LIMMA analyses were applied to predict the prognostic value of LRG1 and its correlation to I-O proteins.

**Results:**

High baseline levels of LRG1 predicted a low frequency of treatment failure in patients undergoing ADT + RT in both the CuPCa and the IMRT cohorts. LRG1 was moderately correlated with CD4, IL6, and CSF1. We identified I-O proteins predicting metastatic failure (MF) at different timepoints.

**Conclusion:**

LRG1 biomarker is associated with I-O proteins and can be used to improve stratification and monitoring of prostate cancer patients undergoing ADT + RT. This work will require further in-depth analyses in independent cohorts with treatment outcome data.

**Graphical abstract:**

Study outline. A) Study cohorts. B) Sampling time points in a longitudinal cohort. 
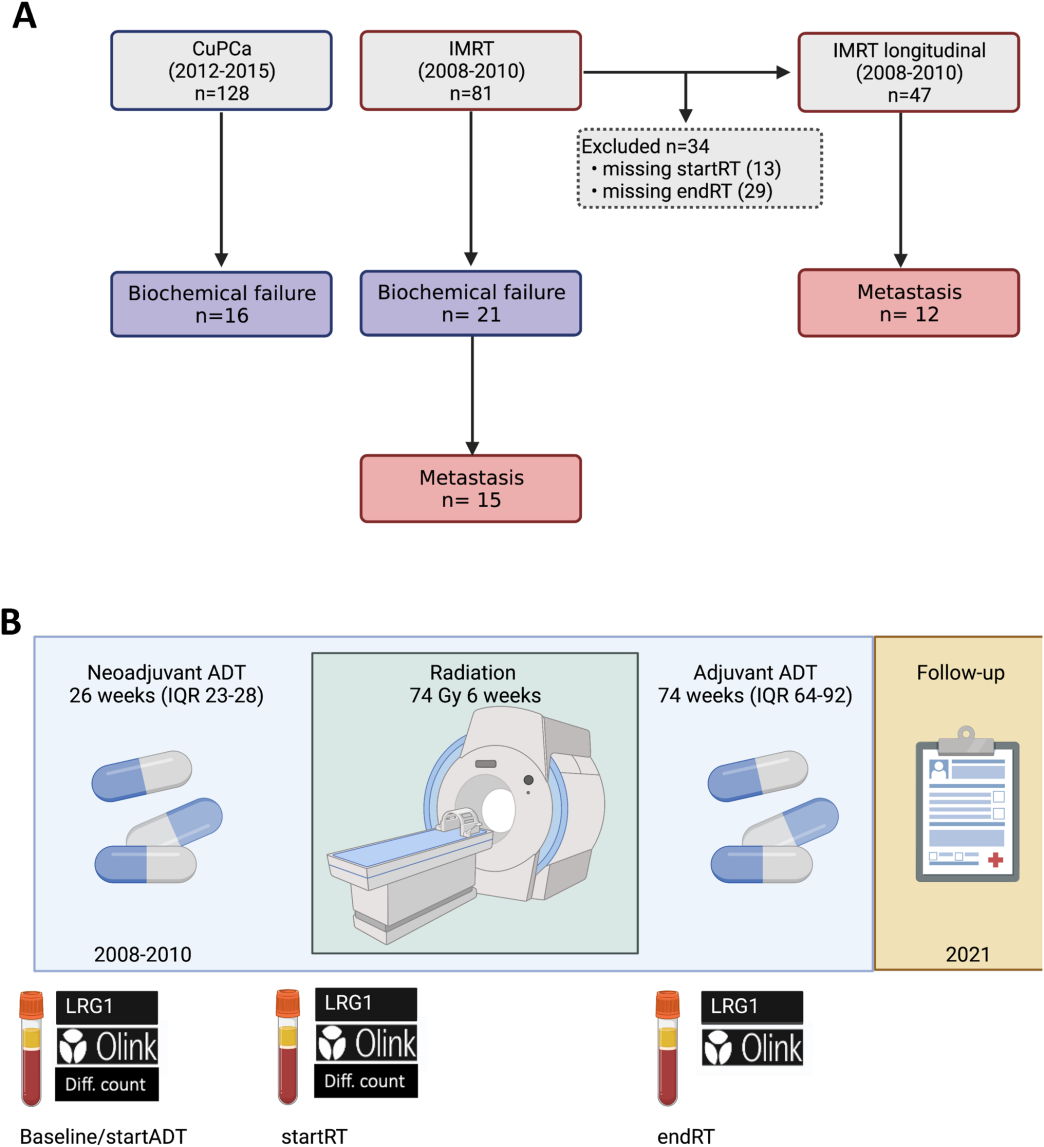

**Supplementary Information:**

The online version contains supplementary material available at 10.1007/s00345-024-04787-8.

## Introduction

Combined androgen-deprivation therapy (ADT) and radiotherapy (RT) provide survival benefits in patients diagnosed with high-risk prostate cancer (PCa) and low-volume metastatic disease [1–3]. Hypotheses have been proposed to explain these benefits based on the knowledge gained from characterizing responses to ADT and RT when used as monotherapies. The androgen receptor (AR) plays a significant role in determining the equilibrium of proliferation, differentiation, survival, and apoptosis in the prostate. It operates through intricate gene transcriptional networks that can be disrupted by external factors like inflammation and oxidative stress, as well as internal factors such as nutrient deficiency and genetic abnormalities. Ablation of androgens through ADT suppresses proliferation and differentiation while inducing growth arrest and cell death [4].

Instead, the cytotoxic effects of RT primarily rely on causing DNA damage directly or through reactive oxygen species (ROS), triggering apoptosis, necrosis, mitotic catastrophe, and/or senescence [5]. ADT also leads to acute hypoxia as androgen-sensitive blood vessels shrink, stimulating the upregulation of pro-angiogenic factors and subsequent neovascularization [4, 6]. Neoadjuvant ADT may thus induce hypoxia and ROS [7]. Interestingly, the AR-axis is also essential in maintaining the DNA integrity in proliferating cells by regulating the transcription of DNA-damage repair genes [8]. This provides an additional potential explanation for the synergistic effects observed when combining ADT and RT.

Recent evidence suggests that combined treatment of ADT and RT elicits a synergistic effect by engaging local and systemic immune responses. This immune response has been proposed to explain the enhanced therapeutic outcome observed when ADT and RT are used together [7]. ADT has been found to recruit anti-tumorigenic T-cell populations initially. However, over time, these anti-tumorigenic cell populations are eventually replaced by pro-tumorigenic immune cells, such as myeloid-derived suppressor cells. This transition in immune cell composition supports the development of androgen-independent PCa [9]. RT, on the other hand, has been shown to promote immunogenic cell death. This occurs through the release of pro-inflammatory cytokines and factors following DNA damage, subsequently leading to the activation of an anti-tumorigenic T-cell response [10, 11]. Interestingly, patients with metastatic PCa who receive the combined treatment of ADT and RT demonstrate improved survival outcomes compared to ADT alone. This suggests the possibility of abscopal immune responses, where localized treatment affects the targeted area and induces systemic immune responses, impacting tumors outside the treated region [12].

We have previously demonstrated earlier BF and castration-resistant disease in patients with low levels of the Leucine-rich alpha-2-glycoprotein 1 (LRG1) before radical prostatectomy [13]. Further, levels of LRG1 are associated with locally advanced disease and early tumor dissemination to the lymph nodes. Patients diagnosed with lymph node metastasis can receive ADT + RT as an adjuvant or salvage treatment. Therefore, it is of great interest to investigate whether the blood levels of LRG1 could serve as a predictive indicator for the outcome of ADT + RT.

LRG1 is a key regulator of myeloid differentiation [14, 15], expressed in most myeloid linages [16], and induced in response to specific acute-phase cytokines [17, 18]. Thus, coupling the dynamics of circulating LRG1 with components of the immune system throughout the treatment schedule of ADT and RT might provide insights into specific conditions that favor complete tumor regression.

## Materials and methods

### Patient cohorts

This study presents patient data from two independent study cohorts (Copenhagen uPAR prostate cancer (CuPCa) and Intensity-Modulated RadioTherapy (IMRT) trial). All patients included were treatment-naïve at inclusion, categorized according to TNM classification 2002, with staging by digital rectal examination (DRE, CUPCa) and magnetic resonance imaging (MRI, IMRT). Biopsies were graded according to the Gleason Score of ISUP 2005 and patients were stratified by D’Amico risk classification. Detailed descriptions of study cohorts are provided in Supplementary Information, and clinicopathological details are shown in Table 1. The study outline is depicted in the Graphical abstract (Supplementary Information). All patients gave written informed consent before inclusion.

**Table 1:** Clinicopathological characteristics study cohorts

	Cohort		
	CuPCa	IMRT (all)	IMRT longitudinal
Total, *n*	128	81	47
Year of enrollment	2012–2015	2008–2010	2008–2010
Follow-up, months, median [IQR]	96 [82–106]	141 [114–149]	136 [104–148]
Age, years, median [IQR]	66 [62–69]	66 [62–70]	66 [62–70]
PSA, ng/ml, median [IQR]	11 [8–21]	26 [15–46]	32 [18–51]
GS, *n* (%)			
3 + 4	44 (34.4)	8 (9.9)	5 (10.6)
4 + 3	24 (18.8)	20 (24.7)	11 (23.4)
4 + 4	27 (21.1)	33 (40.7)	17 (36.2)
≥ 4 + 5	33 (25.8)	21 (25.9)	14 (29.8)
T stage, *n* (%)			
≤ T2c	18 (14.1)	14 (17.3)	6 (12.8)
T3a	62 (48.4)	32 (39.5)	18 (38.3)
T3b/4	48 (37.5)	35 (43.2)	23 (48.9)
CPG, *n* (%)			
4	57 (44.6)	46 (56.8)	24 (51.0)
5	71 (55.5)	35 (43.2)	23 (48.9)
LN + , *n* (%)	10 (13.3)	23 (28.7)	16 (34.0)
BF, *n* (%)	16 (12.5)	30 (33.3)	21 (44.7)
MF, *n* (%)	-	15 (18.5)	12 (25.5)
Mortality, *n* (%)			
Alive	99 (77.3)	56 (69.1)	30 (63.8)
OCM	26 (20.3)	15 (18.5)	10 (21.3)
PCSM	3 (2.3)	10 (12.3)	7 (14.9)

*BF*  biochemical failure, *CPG* Cambridge Prognostic Group, *IQR* interquartile range, *GS* Gleason Score, *LN* Lymph node, *MF* metastatic failure, *OCM* other-cause mortality, *PCSM* prostate cancer-specific mortality

### Blood analysis

Concentrations of LRG1 in plasma and serum were measured by ELISA as previously described [13]. Quantification of 92 immune-oncology-related proteins was performed by Olink. Additional details can be found in Supplementary Information. 

### Statistical analyses

All statistical analyses were performed in R (*version* 4.1.2). Biochemical failure (BF, defined as a rise by 2 ng/mL or more above the nadir PSA) and metastatic failure (MF, radiologically confirmed dissemination into distant sites). BF-free survival was calculated as the time from the start of ADT until the first of, or censored, on the last date PSA measurement was performed. MF-free survival was calculated as the time from start of ADT until the date of radiographically confirmed dissemination into distant sites.

Time-dependent survival estimates were established using Kaplan–Meier and Cox regression models. LRG1 was modeled as a dichotomized marker (high versus low) in survival analyses, with cutoff as previously defined [13]. Multivariable Cox Proportional Hazards modeling with Cambridge Prognostic Group (CPG) as covariate [19] was used to assess the adjusted association of LRG1 with BF and MF. Fine-Gray hazard model was used to test for competing risks. Spearman rank correlation was used to assess associations between single analytes at different timepoints, with Bonferroni correction to account for false discoveries. Fold change differences between groups were estimated using linear models for microarray data (LIMMA).

## Results

### High LRG1 is associated with favorable outcomes in patients undergoing combination treatment of ADT + RT 

**Fig. 1:**
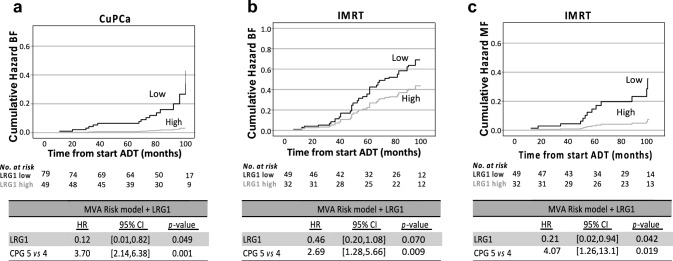
LRG1 predicts risk of progression in men with locally advanced prostate cancer. Cumulative hazard plot with multivariable Cox proportional hazard model coefficients (below) for biochemical failure (BF) in the CuPCa (**a**) and IMRT (**b**), and metastatic failure (MF) in the IMRT cohort (**c**). The cumulative hazard plots are stratified by LRG1 concentration at baseline and adjusted for Cambridge Prognostic Group (CPG)

We initially analyzed plasma samples from 128 patients enrolled in the CuPCa study who had undergone IMRT treatment combined with neoadjuvant ADT. All patients were diagnosed with high-risk and locally advanced PCa, and 16 (12%) experienced BF during the 96-month follow-up period (Table 1). Patients with high LRG1 measured at baseline (treatment-naïve) presented with higher median age and a lower biopsy Gleason Score (Supplementary Table 1). In a Cox regression model, including CPG as a covariate, patients with high baseline LRG1 demonstrated an 88% reduced hazard of developing BF compared to patients with low levels (Fig. 1a).

To validate this observation, we identified a cohort of 81 patients enrolled in the IMRT study at Oslo University Hospital, with a minimum of ten-year follow-up. This study enrolled patients for IMRT with standard neoadjuvant and adjuvant ADT. A greater proportion of these patients were diagnosed with higher Gleason Score, T stage, cancer disseminated into pelvic lymph nodes, and 30 patients experienced BF during the median 12-year follow-up time. In this cohort, patients with low LRG1 at baseline were significantly younger than patients with high levels. No association with other clinicopathological characteristics was observed. Patients with high LRG1 at baseline showed a reduced risk of BF and later radiographic confirmed progression into distant sites, summarized as MF (Supplementary Table 1, and Fig. 1b, c). This was not caused by higher other-cause mortality (Fine-Gray estimation, Supplementary Fig. 1).

### Patients with favorable disease outcomes show consistently high LRG1 levels during treatment

Next, we sought to assess whether blood levels of LRG1 were altered throughout the treatment protocol for these patients. Matched blood samples drawn at start of RT and end of RT were available for 47 of the 81 patients initially included in the IMRT cohort. Patients maintained their LRG1 level throughout the treatment protocol, with minor changes (Supplementary Fig. 2a). Patients with high LRG1 at baseline and end of RT were less likely to experience treatment failure and recurrence with metastatic disease.

### LRG1 associated with high levels of analytes involved in anti-tumor immunity

#### Baseline LRG1 associates with beneficial counts of leukocytes and neutrophils at start of radiotherapy

LRG1 is a protein known to be important in myelopoiesis and neutrophil maturation [15]. Thus, we next wanted to assess whether LRG1 was associated with a higher frequency of leukocytes, neutrophils, and lymphocytes. The frequency of these blood cells was routinely assessed at baseline and start of RT, however, not available at the end of RT. Next, we plotted the counts of the leukocytes, neutrophil, and lymphocyte fractions at baseline and the start of RT based on whether the patients had high or low LRG1 levels at baseline (Supplementary Fig. 2c). Patients with low LRG1 at initiation of ADT had significantly lower leukocyte and neutrophil counts when starting RT. In contrast, patients with high LRG1 at baseline kept stable leukocyte, and neutrophil counts through the same period. This effect was not observed in the number of lymphocytes.

#### Baseline levels of LRG1 associated with cytokines with favorable anti-tumor activities

After observing an association between LRG1, leukocytes, and neutrophils, we next wanted to assess whether LRG1 is associated with proteins of known functions within anti-tumor immunity. Multiplex analysis of 92 analytes was performed by the proprietary Olink technology on samples from all timepoints for the 47 patients, and correlation was assessed by Spearman rank (Supplementary Fig. 2b).

In general, in serum, LRG1 correlated to CD4, CCL23, Gal9, TNFRSF9, and CSF1 from treatment-naïve patients. Only the association with CSF1 was maintained throughout the treatment sequence. LRG1 was also correlated with IL6 at baseline and start of RT, although lost at the end.

Next, we performed LIMMA analyses to assess whether there were differentially expressed cytokines between patients that had high or low LRG1 at baseline and those that would later develop metastasis compared with those that would remain stable or disease-free within 12 years of observation (Supplementary Fig. 3, Supplementary Tables 3–5). LAMP3, IL6, CXCL9, CXCL5, and CCL23 were differentially expressed between LRG1 low and high patients at baseline. MCP1/CCL2 and LAMP3 discriminate the LRG1 groups at start of RT. LAMP3 was also differentially expressed between the LRG1 groups at the end of RT. No overlap between analytes discriminating patients with low or high LRG1 and analytes differentially expressed between patients progressing or not progressing to metastasis was observed at baseline (Supplementary Fig. 3a). LRG1 low patients and those with later metastasis showed significantly lowered levels of the MCP1/CCL2 at start of RT (Supplementary Fig. 3b). Further, at end of RT, patients that would later progress with metastasis and those with low baseline LRG1 showed a distinct lowered concentration of LAMP3 (Supplementary Fig. 3c).

### Discussion

This study shows that patients with high LRG1 experience less BF and MF after ADT + RT. This notion was further explored in association with other immune-related blood analytes in a longitudinal cohort, identifying several novel components that could potentiate the effects of ADT + RT.

Firstly, LRG1 levels at baseline were related to the number of leukocytes and neutrophils at the beginning of RT protocol. ADT affects hematopoiesis [20], and hemoglobin decline during neoadjuvant ADT before RT is an established risk factor for poorer outcomes [21]. LRG1 is shown to antagonize the inhibitory effects of TGFβ1 on colony growth of human CD34^+^ cells and myeloid progenitors [15], and low levels of LRG1 at baseline would be unfavorable for the overall myelopoiesis, including reduced counts of erythrocytes. Neutrophils, another product of myelopoiesis, are an important component in harnessing long-lasting anti-tumor immune responses after radiotherapy [22]. LRG1 was early identified as an important driver of the maturation of neutrophils to granulocytes and is secreted by the secondary granules at the site of inflammation [14]. The elevated levels of LRG1 observed in patients who did not show metastatic failure might have been associated with higher neutrophil activity after RT.

Using the innovative multiplex platform of Olink, we could establish the relationship between 92 immune-oncology-related proteins, levels of LRG1, and metastatic disease outcome. LRG1 levels were associated with the circulatory levels of CSF1 throughout the treatment. CSF1 shows multi-faceted roles that are highly context-dependent. CSF1 plays a key role in hematopoiesis as a growth factor that regulates the proliferation, differentiation, and activation of monocytes and, as such, contributes to the overall pool of monocytes in blood and tissue [23, 24]. Like LRG1, CSF1 is also important in regulating redundant inflammation in the tumor microenvironment and in achieving timely wound healing after chemical and physical insult [25].

Patients with high LRG1 at baseline also demonstrated elevated levels of chemoattractants for neutrophils (CXCL5) and NK cells (CXCL9) [26]. Notably, LRG1 also correlated with a key regulator of neutrophil homeostasis, trafficking, and activity (IL6) and the newly identified neutrophil-secreted chemokine CCL23 [27], both at baseline and start of RT protocol.

Further, assessing differential expression at start of RT, we found the monocyte-attractant protein MCP1/CCL2 to be significantly less expressed in patients that would later recur with metastasis. The protein was also significantly lower in patients with low LRG1. CD4, a co-receptor expressed on T-helper cells, monocytes, macrophages, and dendritic cells (DC), thus is positioned to be a key molecule to propagate optimal anti-tumor activity on several levels [28].

Another protein that predicted favorable outcomes by LIMMA was the LAMP3, a protein exclusively expressed on activated, mature DCs, and has been shown to predict good prognosis in other cancers (e.g., high-grade ovarian) [29]. Patients with high LRG1 at baseline consistently expressed high LAMP3 levels through the treatment protocol. Activated DCs would support the development of long-term memory against tumor antigens shed by irradiated tumor cells.

Although LRG1 levels appeared unaffected throughout the treatment protocol, patients with high LRG1 also expressed other proteins that would serve a purpose in the local clearing of the tumor and incite long-term anti-tumor memory.

Several limitations hamper the conclusion of our study. We found patients with high LRG1 levels at baseline to be experiencing less recurrence and progression in two independent cohorts, high LRG1 is associated with higher age thus, to translate our findings into clinical practice would require prospective studies with age-matched groups. However, using competing risk analysis, we did not find a significant difference between high or low LRG1 patients concerning other-cause mortality.

Further, our study was not powered to establish a clear understanding of the role of LRG1 in propagating the long-term effect of ADT + RT. Functional studies are needed to elucidate how LRG1, alongside other immune-associated factors, can affect the outcome of radiotherapy or other modalities. Nevertheless, we have identified several novel biomarker candidates (i.e., LRG1, TWEAK, and LAMP3) that could form the basis for building a prediction model to screen patients that could show the benefit of combining ADT + RT with androgen-receptor signaling inhibitors, like abiraterone. Likewise, the benefit of neoadjuvant and long-term adjuvant ADT is being discussed for patients with high-risk diseases and proposed that further stratification of these patients could be an option to reduce the overall reduction in quality of life. The small sample size and high variability in analyte levels stalled any validation attempts in our cohort. Nevertheless, this is, to our knowledge, the first study analyzing 92 immune-oncology-related proteins throughout the treatment protocol of ADT + RT in PCa, proposing intriguing suggestions on how ADT + RT could elicit such striking results both on local control but also the effects on metastatic sites. Extrapolating results based on the measurement of circulating analytes into the mechanisms of tumor clearance requires further assessment of immune cell populations in both tumor tissue and tumor-draining lymph nodes where long-term immune education occurs. Furthermore, as the -omics platforms, like Olink, are showing great promise in both feasibilities for high-throughput and standardization, it will be instrumental to demonstrate the practicality in clinical implementation.

### Conclusion

A high baseline level of the immune-associated protein LRG1 is favorable in patients undergoing ADT + RT. Patients with elevated levels of CD4 and CXCL12 at the time of starting RT protocol show prolonged treatment responses, together with the LAMP3. LRG1, CD4, and CXCL1 inform the need for adjuvant treatment modalities in the immunotherapy era.

## Supplementary Information

Below is the link to the electronic supplementary material.

Supplementary file1 (DOCX 32 KB)

Supplementary file2 (PDF 247 KB)

Supplementary file3 (DOCX 28 KB)

## Data Availability

Data can be made available upon request to the corresponding author.
