# Supplementary material for: Systemic interrogation of immune-oncology-related proteins in patients with locally advanced prostate cancer undergoing androgen deprivation and intensity-modulated radiotherapy
Source: World J Urol. 2024 Feb 22;42(1):95. doi: 10.1007/s00345-024-04787-8 (PMC10884049; doi:10.1007/s00345-024-04787-8)
Supplement: Supplementary file 1 — Supplementary file1 (DOCX 32 KB) [file 345_2024_4787_MOESM1_ESM.docx]

| **Table S1. Clinicopathological characteristics stratified by LRG1 concentrations** | | | |  | | |
| --- | --- | --- | --- | --- | --- | --- |
|  | **CuPCa cohort** | | | **IMRT cohort** | | |
|  | **LRG1 low** | **LRG1 high** | ***p*-value** | **LRG1 low** | **LRG1 high** | ***p*-value** |
| *n* | 79 | 49 |  | 49 | 32 |  |
| Age, years, median [IQR] | 67 [63, 69] | 69 [66, 71] | 0.029 | 63 [59, 69] | 67 [64, 72] | 0.028 |
| PSA, ng/mL, median [IQR] | 11.6 [8.1, 23.5] | 11.0 [7.5, 18.0] | 0.999 | 31.0 [15.3, 48.0] | 26.0 [14.0, 44.0] | 0.595 |
| GS, *n* (%) |  |  | 0.001 |  |  | 0.765 |
| 3+4 | 18 (22.8) | 26 (53.1) |  | 5 (10.2) | 3 (9.4) |  |
| 4+3 | 14 (17.7) | 10 (20.4) |  | 11 (22.4) | 9 (28.1) |  |
| 4+4 | 19 (24.1) | 8 (16.3) |  | 19 (38.8) | 14 (43.8) |  |
| ≥4+5 | 28 (35.4) | 5 (10.2) |  | 14 (28.5) | 6 (18.8) |  |
| T stage, *n* (%) |  |  | 0.417 |  |  | 0.827 |
| ≤T2c | 8 (10.1) | 8 (17.0) |  | 10 (20.4) | 4 (12.6) |  |
| T3a | 40 (50.6) | 22 (46.8) |  | 17 (34.7) | 15 (46.9) |  |
| ≥T3b | 31 (39.2) | 17 (36.2) |  | 22 (44.9) | 13 (40.6) |  |
| LN+, *n* (%) | 7 (8.9) | 3 (6.1) | 0.478 | 13(26.5) | 10 (31.3) | 0.425 |
| CPG, *n* (%) |  |  | 0.058 |  |  | 0.701 |
| ≤4 | 30 (38.0) | 27 (55.1) |  | 27 (55.1) | 19 (59.4) |  |
| 5 | 49 (62.0) | 22 (44.9) |  | 22 (44.9) | 13 (40.6) |  |
| BF, *n* (%) | 16 (20.3) | 1 (2.0) | 0.003 | 22 (44.9) | 8 (25) | 0.070 |
| MF, *n* (%) |  |  |  | 12 (24.5) | 2 (6.3) | 0.034 |
| PCSM |  |  |  | 7 (14.3) | 3 (9.4) | 0.580 |
| OCM, *n* (%) | 9 (11.4) | 15 (30.6) | 0.009 | 7 (14.3) | 8 (25.0) | 0.572 |
| Abbreviations: BF = biochemical failure; ClinProg = Clinical Progression; CPG = Cambridge Prognostic Group; GS = Gleason Score; IQR = interquartile range; LN = Lymph node; OCM = other-cause mortality; PCSM = prostate cancer specific mortality. | | | | | | |

| **Table S2. Clinicopathological characteristics of patients in the IMRT longitudinal cohort stratified by serum LRG1 concentrations at baseline.** | | | |
| --- | --- | --- | --- |
|  | **LRG1 low** | **LRG1 high** | ***p*-value** |
| *n* | 32 | 15 |  |
| Age, years (median [IQR]) | 63.13 [57.89, 67.58] | 66.22 [63.85, 70.93] | 0.055 |
| PSA, ng/mL, (median [IQR]) | 32.50 [18.75, 52.73] | 31.00 [15.00, 41.50] | 0.584 |
| GS, *n* (%) |  |  | 0.292 |
| 3+4 | 2 (6.2) | 3 (20.0) |  |
| 4+3 | 7 (21.9) | 4 (26.7) |  |
| ≥4+4 | 23 (71.9) | 8 (53.3) |  |
| T stage, *n* (%) |  |  | 0.276 |
| ≤T2c | 4 (12.4) | 2 (13.3) |  |
| T3a | 10 (31.2) | 8 (53.3) |  |
| ≥T3b | 18 (56.2) | 5 (33.3) |  |
| LN+, *n* (%) | 10 (31.2) | 6 (40.0) | 0.285 |
| CPG, *n* (%) |  |  | 0.068 |
| ≤4 | 13 (40.6) | 11 (73.3) |  |
| 5 | 19 (59.4) | 4 (26.7) |  |
| BF, *n* (%) | 16 (50.0) | 5 (30.0) | 0.057 |
| MF, *n* (%) | 11 (34.4) | 1 (6.7) | 0.043 |
| Abbreviations: BF = biochemical failure; ClinProg = Clinical Progression; CPG = Cambridge Prognostic Group; GS = Gleason Score; IQR = interquartile range; LN= lymph node (stage) MF = metastatic failure; OCM = other-cause mortality; PCSM = prostate cancer specific mortality | | | |

| **Table S3. Relationship between LRG1 and immune- and oncology related proteins in blood sampled at different time points during treatment, Ranked by p-value,** | | | | | |
| --- | --- | --- | --- | --- | --- |
| **Time point** | **Analyte** | **Spearman, *rho*** | ***p*-value** | **Pearson, r** | ***p*-value** |
| Baseline | CD4 | 0,57 | <0,001 | 0,49 | <0,001 |
|  | IL6 | 0,37 | 0,01 | 0,57 | <0,001 |
|  | CCL23 | 0,47 | 0,001 | 0,47 | 0,001 |
|  | Gal9 | 0,47 | 0,001 | 0,39 | 0,007 |
|  | TNFRSF9 | 0,43 | 0,003 | 0,38 | 0,009 |
|  | CSF1 | 0,4 | 0,006 | 0,37 | 0,01 |
|  | LAMP3 | 0,39 | 0,007 | 0,31 | 0,035 |
|  | PDL1 | 0,36 | 0,012 | 0,33 | 0,025 |
|  | VEGFA | 0,34 | 0,018 | 0,33 | 0,026 |
|  | CCL4 | 0,34 | 0,018 | 0,3 | 0,038 |
|  | TRAIL | 0,36 | 0,013 | 0,3 | 0,04 |
| startRT | IL6 | 0,51 | <0,001 | 0,57 | <0,001 |
|  | CSF1 | 0,47 | 0,001 | 0,48 | 0,001 |
|  | ICOSLG | −0,34 | 0,02 | −0,41 | 0,004 |
|  | HGF | 0,29 | 0,046 | 0,4 | 0,005 |
|  | CD4 | 0,34 | 0,019 | 0,37 | 0,01 |
|  | ANGPT2 | 0,32 | 0,03 | 0,37 | 0,01 |
|  | IL2 | −0,33 | 0,023 | −0,34 | 0,018 |
|  | Gal9 | 0,3 | 0,044 | 0,28 | 0,054 |
| endRT | CSF1 | 0,44 | 0,002 | 0,46 | 0,001 |
|  | PGF | 0,4 | 0,005 | 0,29 | 0,05 |
|  | CD83 | 0,4 | 0,006 | 0,35 | 0,015 |
|  | CX3CL1 | 0,39 | 0,007 | 0,35 | 0,017 |
|  | Gal9 | 0,34 | 0,018 | 0,29 | 0,046 |
|  | KIR3DL1 | −0,30 | 0,041 | −0,32 | 0,03 |
|  | NCR1 | 0,42 | 0,003 | 0,27 | 0,069 |
|  | CD27 | 0,31 | 0,037 | 0,22 | 0,142 |
|  | TIE2 | 0,3 | 0,039 | 0,23 | 0,112 |

| **Table S4. Fold change differences in analytes at baseline ADT stratified by LRG1 levels high versus low.** | | | | |
| --- | --- | --- | --- | --- |
| **Time point** | **Analyte** | **logFC** | ***p*-value** | ***Adj. p*-value** |
| Baseline | CD4 | -0.31 | 0.000 | 0.019 |
|  | LAMP3 | -0.71 | 0.001 | 0.019 |
|  | MMP7 | -0.30 | 0.002 | 0.056 |
|  | CCL23 | -0.40 | 0.005 | 0.089 |
|  | IL6 | -0.51 | 0.011 | 0.154 |
|  | MCP1 | -0.37 | 0.012 | 0.154 |
|  | Gal9 | -0.24 | 0.016 | 0.154 |
|  | TNFRSF9 | -0.29 | 0.017 | 0.154 |
|  | TRAIL | -0.19 | 0.018 | 0.154 |
|  | CD83 | -0.24 | 0.019 | 0.154 |
|  | CSF1 | -0.15 | 0.020 | 0.154 |
|  | MCP3 | -0.34 | 0.028 | 0.191 |
|  | CXCL9 | -0.49 | 0.029 | 0.191 |
|  | CD5 | -0.21 | 0.041 | 0.247 |
|  | PDL1 | -0.20 | 0.044 | 0.247 |
|  | CXCL5 | -0.42 | 0.048 | 0.247 |
|  | CCL3 | -0.27 | 0.048 | 0.247 |
| startRT | TRAIL | -0.24 | 0.003 | 0.156 |
|  | CD4 | -0.21 | 0.008 | 0.234 |
|  | MMP7 | -0.25 | 0.010 | 0.234 |
|  | MCP1 | -0.41 | 0.022 | 0.398 |
|  | IL18 | -0.30 | 0.028 | 0.430 |
|  | LAMP3 | -0.41 | 0.035 | 0.461 |
|  | ANGPT2 | -0.29 | 0.041 | 0.474 |
| endRT | MMP7 | -0.28 | 0.003 | 0.136 |
|  | LAMP3 | -0.59 | 0.005 | 0.152 |
|  | CD4 | -0.20 | 0.012 | 0.218 |
|  | HO1 | -0.31 | 0.012 | 0.218 |
|  | IL18 | -0.32 | 0.014 | 0.218 |
|  | ADA | -0.24 | 0.022 | 0.286 |
|  | TRAIL | -0.19 | 0.027 | 0.307 |
|  | TNF | -0.30 | 0.039 | 0.368 |
|  | Gal1 | -0.13 | 0.040 | 0.368 |
|  | CSF1 | -0.11 | 0.044 | 0.368 |

Abbreviations: Adj = Adjusted; logFC = logarithmic fold change

| **Table S5. Fold change differences in analytes at baseline stratified by metastasis status at follow-up. Ranked by *p*-value.** | | | | |
| --- | --- | --- | --- | --- |
| **Time point** | **Analyte** | **logFC** | ***p*-value** | **Adj. *p*-val** |
| Baseline | TWEAK | 0.29 | 0.015 | 0.844 |
|  | ADA | -0.29 | 0.026 | 0.844 |
|  | IL12RB1 | 0.26 | 0.041 | 0.844 |
|  | GZMA | 0.24 | 0.049 | 0.844 |
| starRT | CCL3 | -0.35 | 0.015 | 0.731 |
|  | IL15 | -0.22 | 0.017 | 0.731 |
|  | CXCL12 | -0.21 | 0.027 | 0.731 |
|  | MCP1 | -0.39 | 0.039 | 0.731 |
|  | MCP4 | -0.38 | 0.040 | 0.731 |
| endRT | CXCL5 | -0.51 | 0.016 | 0.799 |
|  | LAMP3 | -0.52 | 0.024 | 0.799 |
|  | TWEAK | 0.22 | 0.046 | 0.799 |

Abbreviations: Adj = Adjusted; logFC = logarithmic fold change
