# Supplementary material for: Systemic interrogation of immune-oncology-related proteins in patients with locally advanced prostate cancer undergoing androgen deprivation and intensity-modulated radiotherapy
Source: World J Urol. 2024 Feb 22;42(1):95. doi: 10.1007/s00345-024-04787-8 (PMC10884049; doi:10.1007/s00345-024-04787-8)

Legends

Supplementary Fig. 1 Cumulative risk for metastatic failure versus other cause mortality (OCM) as estimated by Fine-Gray competing risks estimation.

Supplementary Fig. 2 Longitudinal expression of LRG1 and relation to immune-oncology (IO) related proteins and immune cell populations **A)** Boxplot of LRG1 concentrations in matched serum samples at start ADT (baseline), start radiotherapy (start RT) and end radiotherapy (endRT) grouped by metastatic status at last follow-up. Asterix denotes $p<0.05$ by Mann-Whitney U test. **B)** Line plots of the number of leukocytes, neutrophils, and lymphocytes measured by differential blood count at start ADT (baseline) and startRT*,* grouped by LRG1 level at baseline (high vs low). The significance of the difference in blood count was determined using the Mann-Whitney *U* test. **C)** I-O proteins significantly correlated with LRG1 and with a Spearman rho value of ≥ 0.4 at baseline, start startRT, and at endRT.

Supplementary Fig. 3 Immuno-oncology analytes associated with LRG1 and/or metastatic failure during treatment Interleaved scatterplot illustrating the fold change (FC)) in analyte levels between patients with later metastasis *vs* no metastasis or LRG1 low *vs* high in **A)** samples from start neoadjuvant ADT (baseline)*,* **B)** end neoadjuvant ADT and start radiotherapy (start RT) and **C)** at end radiotherapy (endRT)**.** LIMMA was used to determine the fold change and significance between the groups and analytes significantly different between the groups (unadjusted) LRG1 low and high or metastasis versus no-metastasis are shown. Analytes significant by LRG1 are illustrated on a blue background and metastasis on a red background. Analytes differentially expressed both between LRG1 low and high patients and differentially expressed between patients progressing or not-progressing to metastatic disease are denoted in bold with an asterisk.

Supplementary Fig. 1

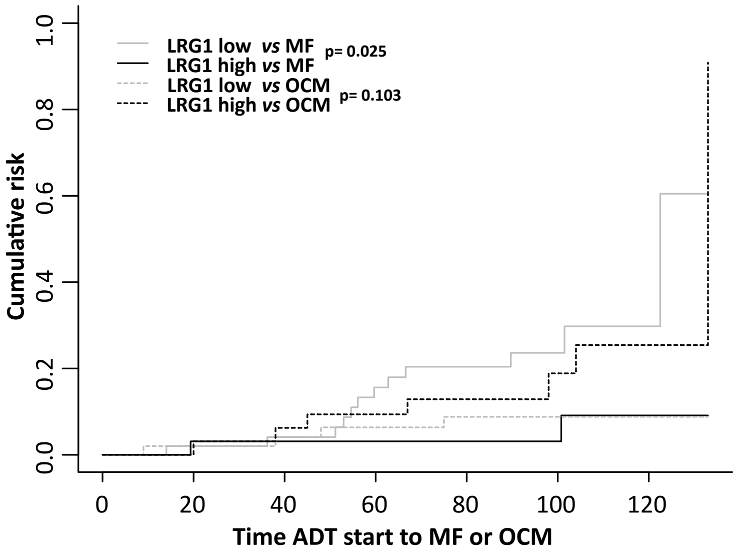

Supplementary Fig. 2

a

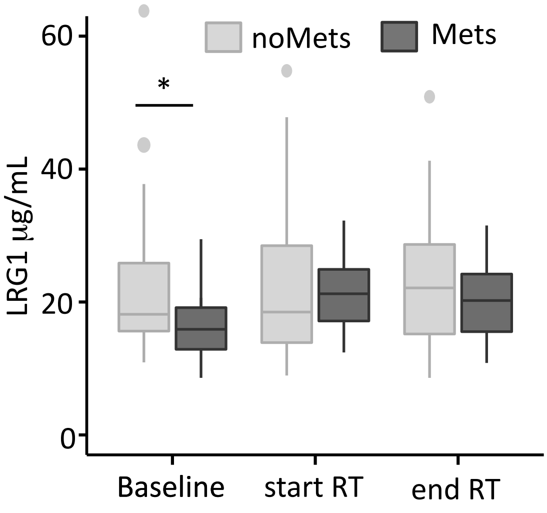

b

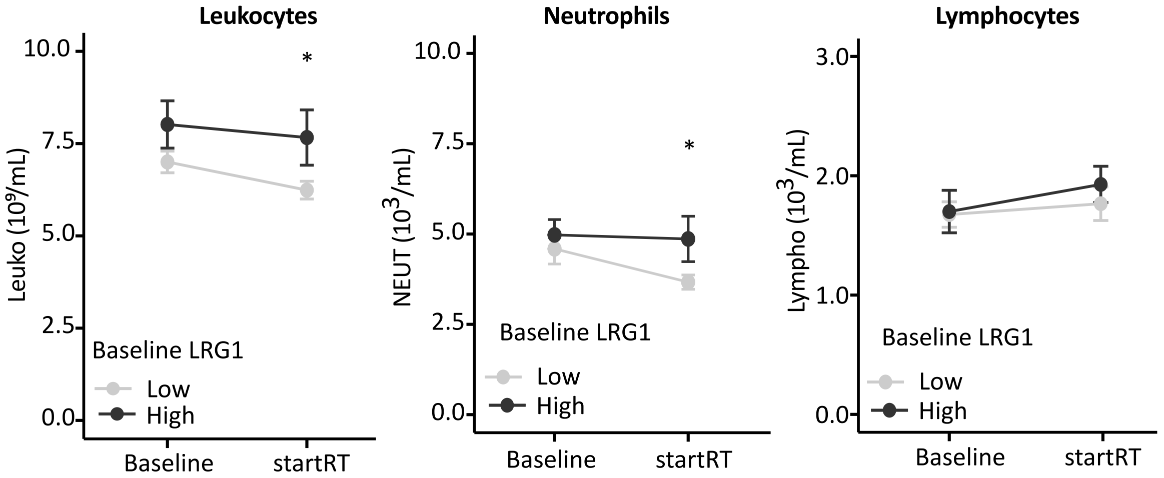

c

Baseline			startRT			endRT		
**I-O analyte**	**Spearman, rho**	**p-value**	**I-O analyte**	**Spearman, rho**	**p-value**	**I-O analyte**	**Spearman, rho**	**p-value**
CD4	0,57	<0,001	IL6	0,51	0,000	CSF1	0,44	0,002
CCL23	0,47	0,001	CSF1	0,47	0,001	NCR1	0,42	0,003
Gal9	0,47	0,001				PGF	0,40	0,005
TNFRSF9	0,43	0,003				CD83	0,40	0,006
CSF1	0,40	0,006				CX3CL1	0,39	0,007
LAMP3	0,39	0,007						
IL6	0,37	0,010						
PDL1	0,36	0,012						
TRAIL	0,36	0,013						

Supplementary Fig. 3

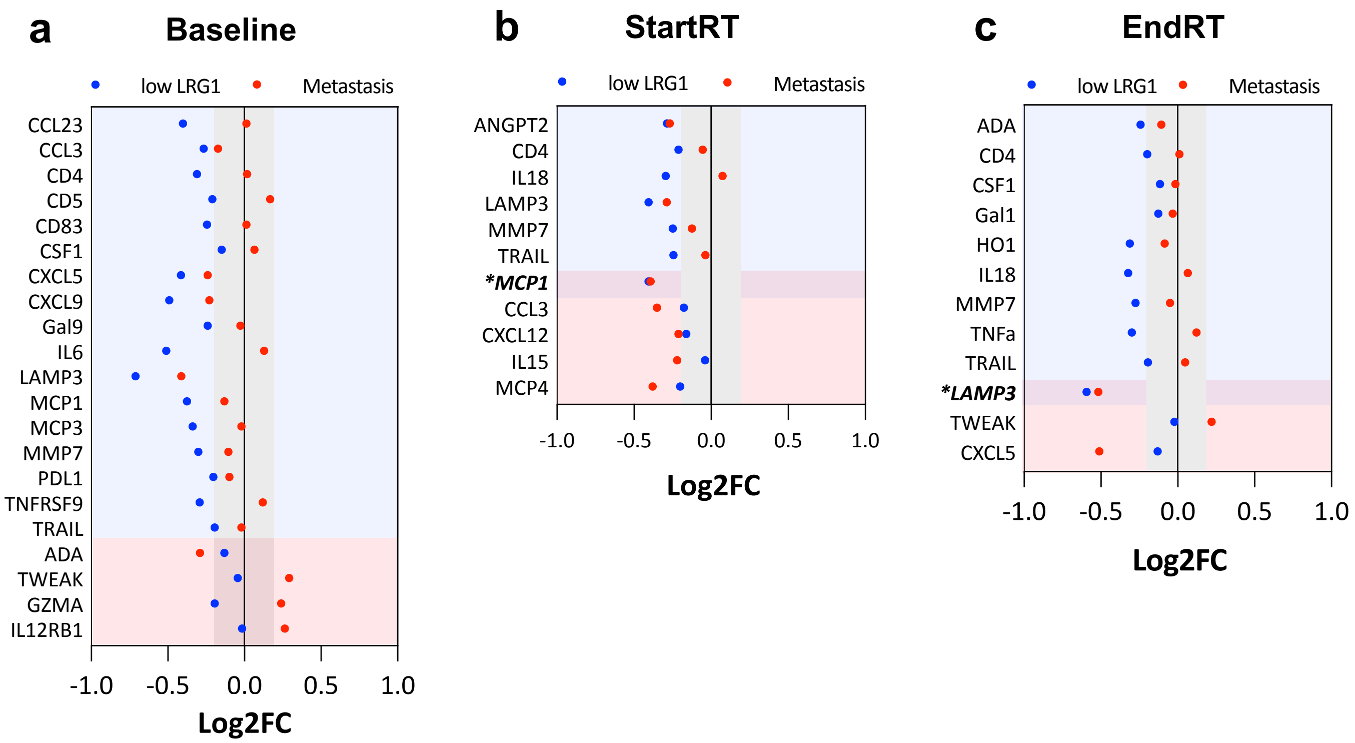

Supplement: Supplementary file 2 — Supplementary file2 (PDF 247 KB) [file 345_2024_4787_MOESM2_ESM.pdf]
