# Supplementary material for: Systemic interrogation of immune-oncology-related proteins in patients with locally advanced prostate cancer undergoing androgen deprivation and intensity-modulated radiotherapy
Source: World J Urol. 2024 Feb 22;42(1):95. doi: 10.1007/s00345-024-04787-8 (PMC10884049; doi:10.1007/s00345-024-04787-8)
Supplement: Supplementary file 3 — Supplementary file3 (DOCX 28 KB) [file 345_2024_4787_MOESM3_ESM.docx]

**Supplementary Information**

**Title manuscript: “Systemic interrogation of immune-oncology related proteins in patients with locally advanced prostate cancer undergoing androgen deprivation and intensity-modulated radiotherapy”**

**Supplementary Material and Methods**

## **Patient cohorts**

### All patients included in this study have been categorized according to the TNM classification 2002, with staging by digital rectal examination (DRE, CuPCa) and magnetic-resonance imaging (MRI, IMRT). Biopsies have been graded according to the Gleason Score of ISUP 2005. Patients have been further stratified by D’Amico risk classification.

### **CuPCa**

The Copenhagen uPAR prostate cancer (CuPCa) study was performed at the Department of Urology, Rigshopitalet Copenhagen, in 2012-2015[1].

In this study, patients with localized and locally advanced PCa with lymph node-negative status were offered image-guided intensity-modulated radiotherapy (IMRT) with 2 Gy × 39 combined with 6 months neo-adjuvant androgen-deprivation therapy (ADT ; Luteinizing hormone-releasing hormone (LHRH) analogs) and further adjuvant ADT up to a total of 36 months for the curative intent. All D’Amico high-risk patients underwent staging lymphadenectomy before radiotherapy and radiation fields were extended per the presence of positive lymph nodes. Baseline (treatment-naïve) plasma samples drawn from 128 participants were included in our current study cohort. The collection of clinical follow-up data was performed on medical records up until September 2021.

### **IMRT**

The Intensity-Modulated RadioTherapy (IMRT) trial was conducted at Oslo University Hospital from 2008-2010 [2]. Of the 90 patients included in the trial, baseline (treatment-naïve) serum samples were available from 81 study participants and were included in the current study. This trial included patients with D’Amico high-risk PCa at diagnosis, with a 15% risk of disseminated disease into pelvic lymph nodes[3]. All patients received 6 months ADT (LHRH analog; Goserelin) before starting radiotherapy protocol, with 2 Gy × 37 targeting the prostate, seminal vesicles, and 23-25 Gy to the nodal basins. Clinical follow-up data were collected until December 2021.

### **IMRT longitudinal cohort**

Matched serum samples from baseline (start neo-adjuvant ADT), start of RT protocol and end of RT protocol were available from 47 of the patients enrolled into the IMRT trial, and were used to study longitudinal alterations in blood analytes.

## **Laboratory analyses**

### **ELISA**

Concentrations of LRG1 in plasma and serum were measured as previously described[4]. Samples from the CuPCa cohort were analyzed at the Central Laboratory of Rigshospitalet, Copenhagen, Denmark, using the fully automated BEP 2000 robotic system from Siemens Healthineers.

Samples drawn from the IMRT study were analyzed at the Hormone laboratory of Oslo University Hospital, Norway. Here, samples were equilibrated at room temperature for 30 minutes before dilution into EIA buffer by serial dilution (serum, 1:3125). Samples were calibrated in EIA buffer for 30 minutes before loading into plate wells. Absorbance was read using a 1420 Multilabel counter, Victor^3^ plate reader (PerkinElmer). Ten plasma samples were included to control for inter-lab variability and used to establish a scaling factor.

### **Olink**

One microliter of full serum was analyzed using Proximity Extension Assay (PEA) technology (olink.com), with a targeted focus panel comprising 92 immune and oncology-related proteins (Immuno-oncology panel V.1). Protein levels were intensity normalized to adjust for batch effect.

### **Ethics**

The CuPCa study was approved by the Danish National Committee on Biomedical Research Ethics for the Capital Region (no.: H-4–2011–071), while the IMRT study was approved by the Norwegian Regional Committees for Medical and Health Research Ethics (REC): REC South East no. 2010/1790. The reporting of clinicopathological variables, survival data, and biomarker expressions was conducted following the REMARK guidelines.

**References**

[1] Lippert S, Berg KD, Hoyer-Hansen G, Lund IK, Iversen P, Christensen IJ, et al. Copenhagen uPAR prostate cancer (CuPCa) database: protocol and early results. Biomark Med. 2016;10:209-16.

[2] Lilleby W, Stensvold A, Dahl AA. Intensity-modulated radiotherapy to the pelvis and androgen deprivation in men with locally advanced prostate cancer: a study of adverse effects and their relation to quality of life. Prostate. 2013;73:1038-47.

[3] Cagiannos I, Karakiewicz P, Eastham JA, Ohori M, Rabbani F, Gerigk C, et al. A preoperative nomogram identifying decreased risk of positive pelvic lymph nodes in patients with prostate cancer. J Urol. 2003;170:1798-803.

[4] Guldvik IJ, Braadland PR, Sivanesan S, Ramberg H, Kristensen G, Tennstedt P, et al. Low Blood Levels of LRG1 Before Radical Prostatectomy Identify Patients with High Risk of Progression to Castration-resistant Prostate Cancer. Eur Urol Open Sci. 2022;45:68-75.
